# Investigation the Role of Autophagy in Non-Small Cell Lung Cancer

**DOI:** 10.31557/APJCP.2021.22.3.947

**Published:** 2021-03

**Authors:** Minoo Pargol, Shima Zare Karizi, Masoumeh Akbari, Bahareh Nourmohammadi, Mohammad Behgam Shadmehr, Morteza Karimipoor, Shohreh Zare Karizi

**Affiliations:** 1 *Department of Genetics and Biotechnology, School of Biological Science, Varamin Pishva Branch, Islamic Azad University, Varamin, Iran. *; 2 *Molecular Medicine Department, Biotechnology Research center, Pasteur Institute of Iran, Tehran, Iran. *; 3 *Tracheal Diseases Research Center, National Research Institute of Tuberculosis and Lung Diseases Shahid Beheshti University of Medical Sciences, Tehran, Iran. *

**Keywords:** MicroRNAs, non-small cell lung cancer, autophagy, DNA methylation

## Abstract

**Objective:**

Recent studies have shown the role of autophagy in different types of cancer including lung cancer. MicroRNAs are considered as key factors in regulation of autophagy related genes. miR-30d, miR-204-5p and miR-20a are regulatory markers which can suppress the expression of beclin1, LC3, bcl2 and ULK1 as their target genes and they lead to decrement of autophagy in human cancer cells. Moreover, epigenetic modifications DNA methylation has been indicated in regulation of autophagy in different stages of cancer.

**Methods:**

In this study, the expression levels of miR-30d, miR-204-5p and miR-20a as well as their target genes were analyzed in 30 non-small cell lung cancers (NSCLCs) patients sample and adjacent normal tissues by real-time qPCR. In addition, DNA methylation of beclin1, LC3, bcl2 and ULK1 genes were assessed by MS-HRM method.

**Results:**

MiR-30d (p value= 0.01) and miR-204-5p (P=0.048) significantly down-regulated in tumor samples compared to normal adjacent tissues, while there was no significant change in expression level of miR-20a. On the other hand, target genes expression level was significantly increased in NSCLC tissues, however methylation pattern of the target gene promoters, did not show any significant alteration.

**Conclusion:**

These results indicate roles for miR-30d, miR-204-5p as tumor suppressor genes as well as target genes as oncogenes in NSCLC patients. Although these factors may have a significant role in NSCLC progression, further studies are necessary to investigate the implications of these findings for treatment of lung cancer.

## Introduction

Lung cancer is the leading cause of cancer related death worldwide. Non-small cell lung cancer (NSCLC) accounts for approximately 85% of all lung cancer cases. NSCLC is histologically classified to three main subtypes: squamous cell carcinoma (SCC), large cell carcinoma (LCC) and adenocarcinoma (ADC) (Hou et al., 2010). The last two subtypes, compromises 25–30% and 40% of all NSCLC patients, respectively. The prognosis of lung cancer remains poor and despite various progresses in diagnostic and therapeutic processes, the 5-year survival has only increased from 15.7% to 17.4% during the last decade (Boolell et al., 2015). 

Macro-autophagy (hereafter referred to as autophagy), which is a homeostatic mechanism regulating degradation of proteins, organelles, and other cellular components in lysosomes, plays an important role in cancer initiation, progression and development (Frankel and Lund, 2012). It is an evolutionary conserved phenomenon observed in all eukaryotic cells, from yeast to mammals involving in turnover of proteins and organelles as well as adaptation and survival, or death in different environmental unfavorable conditions such as nutrient deprivation and pathogen infection (Kondo et al., 2005). Therefore, malfunction of this catabolic pathway contributes to various human diseases including cancer (Frankel et al., 2011).

Approximately 30 different genes have been studied to be involved in regulating autophagy (Wang et al., 2015). Beclin1, the mammalian orthologue of yeast Atg6, participates in autophagosome formation by interacting with type III phosphatidylinositol 3-kinase (PI(3)KC3) complex (Takahashi et al., 2007). Beclin1 possesses a conserved domain (ECD), mediating its interaction with PI(3)KC3–Vps34, as well as bcl2homology3 (BH3) domain that regulates its binding to bcl2 family (particularly bcl2 and its homologue BclXL) (Maiuri et al., 2007). Bcl-2 (B-cell lymphoma/leukemia-2), the antiapoptotic protein, suppresses the autophagic activity of the Beclin1–PI(3)KC3 complex via its binding to Beclin1 during non-starvation conditions (He and Klionsky, 2009). Microtubule associated protein light chain 3 (LC3), the mammalian counter part of yeast Atg8, also plays a pivotal role in autophagosome formation (Jiang et al., 2012). LC3 includes soluble and membrane-bound forms, LC3 I and LC3 II respectively (Miracco et al., 2010). Various types of stressors induce the conversion of LC3-I to LC3-II, which has been considered to be the most reliable autophagosomal marker (Jiang et al., 2012). UNC-51-like kinase 1 (ULK1), the homologue of Atg1 in mammals, is a multifunctional protein that regulates initial events of autophagy signaling (Chen et al., 2014).

MicroRNAs (miRNAs) are small non-coding RNA molecules that regulate the expression of their target genes at post-transcriptional level. They have essential roles in controlling various cellular activities including differentiation, proliferation, apoptosis and tumorigenesis. Several studies indicate that miRNAs are also involved in the regulation of autophagy. Inhibition of some autophagic proteins by miRNAs has been suggested to hinder the progression of lung cancer (Liu and Huang, 2015). *miR-30*d, member of *mir-30* miRNA family, regulates numerous genes in autophagy process including* beclin1*. It has been shown that *miR-30d* suppresses the expression of* beclin1* gene, which leads to decreasing autophagy in human cancer cells (Yang et al., 2013; Zhao et al., 2017a). MiR-204-5p (previously named as miR-204 before miRBase release 19.0) shows significantly reduced expression and exerts its function as a tumor suppressor in several cancers such as glioblastoma, prostate cancer and NSCLC (Luan et al., 2017). It has also been determined miR-204-5p is related to autophagy pathway in various types of cancers and increased function of LC3-II in autophagy is inhibited by increasing miR-204-5p level in colorectal cancer (Sümbül et al., 2014). Bcl-2 is also the functional target of *miR-204-5p*, which decreases its protein expression by targeting 3’-untranslated region (UTR) of bcl-2 (Sacconi et al., 2012). MiR-20a is a member of the miR-17-92 cluster, which has been widely studied to have oncogenic function. MiR-20a has been shown to regulate autophagy pathway negatively in breast cancer cells. Moreover, inhibition of leucine deprivation–induced autophagy via down-regulation of ULK1 by miR-20a has been demonstrated in C2C12 myoblasts (Guo et al., 2016). 

DNA methylation is a significant epigenetic mechanism that happens by addition of a methyl group to the cytosine, mostly on CpG islands in promoter region, contributes to the silencing of gene transcription (Xu et al., 2018). Moreover, epigenetic modifications, including silencing of various significant genes by DNA methylation, have been demonstrated in regulation of autophagy in different stages of cancer. Aberrant promoter methylation of beclin1 gene leads to downregulation of its protein in breast cancer tissues. Methylation condition of autophagic genes may play fundamental role in cancer progression. Therefore, understanding novel epigenetic functions in autophagy regulation may contribute to outstretch strategies for cancer diagnosis and treatment (Sui et al., 2015). 

The aim of present study was to evaluate the methylation and expression levels of genes involved in autophagy process and the related miRNAs in NSCLC samples.

## Materials and Methods

In this study, 30 specimens from tumoral tissues and 30 specimens from the adjacent normal tissues were taken from the resected lung lobes in patients who underwent curative surgical resection (lobectomy) for NSCLC from 2011 to 2014 at Masih Daneshvari Hospital (Tehran, Iran). The patients did not receive any preoperative chemotherapy or radiotherapy.

The pathological characteristics of all samples were obtained according to standard protocols from pathologists. The clinico-pathological features of the patients are summarized at Table 1. The study was approved by the ethics committee (sbmu l. REC.1390. 65) of the Masih Daneshvari Hospital and written informed consent was taken from all patients before surgical resection. Fresh tissue samples were transferred immediately to liquid nitrogen and stored at -80°C until use. 

*RNA extraction and cDNA synthesis*

Total RNA was extracted from tumoral and adjacent normal tissues by using Tripure Isolation Reagent (Qiagen, USA) according to the manufacturer’s instruction. The purity and concentration of extracted RNA was measured by Nanophotometer. For cDNA synthesis, mature miRNA was reverse transcribed using specific stemloop primers (Reverted First Strand cDNA Synthesis Kit, Thermo, Lithuania). In brief, 1,000 ng RNA of each sample, 15 pmol stem loop primer, 4 μl 5x buffer, 2 μl dNTP, 0.5 μl Ribo Lock RNAse inhibitor and 0.5 μl reverse transcriptase enzyme were added in total volume of 20 μl. The thermal cycling condition for the cDNA synthesis was as follows: 42°C for 60 min followed by 70°C for 5 min. For cDNA synthesis of target genes, total RNA was reversely transcribed into cDNA using Random hexamer primers (RevertAid First Strand cDNA Synthesis Kit, Thermo, Lithuania). In summary, 1,000 ng RNA of each sample, 10 pmol Random hexamer primers, 4μl 5x buffer, 0.5 μl Ribo Lock RNAse inhibitor, 2μl dNTP, and o.5 μl reverse transcriptase enzyme were added in the total volume of 20 μl. The thermal cycling condition for the cDNA synthesis was as follows: 25°C for 5 min and 42°C for 60 min followed by 70°C for 5 min. 

*Primer design and qRT- PCR*

Gene-specific primers for target genes and also forward and universal reverse primers for miRNAs were designed using GeneRunner and Allele ID 6.0 softwares (Tables 2 and 3). In this study, for detection of *miRNA* expression level as well as target genes in tumor and adjacent normal tissues, SYBER qRT-PCR was performed. All steps were implemented according to the protocols and all samples were performed in duplicate in MicroAmp optical 96-well plate (StepOne plus Applied Biosystem USA). The reactions were normalized with RNU44 control for miRNAs and GAPDH for target genes. In brief, for detection of expression pattern of *miR-20a, miR-30d* and *miR-204-5p* each reaction contains 10 μl SYBR master mix, 5 pmol of specific forward and universal reverse primers and 2 μl of cDNA in total volume of 20μl reaction. The thermal cycling condition for the amplification of mentioned miRNAs were as follows: initial denaturation at 95°C for 15 min, followed by 40 cycles of 95°C for 20 s and 60°C for 1 min for miR-20a and miR-204-5p as well as initial denaturation at 95°C for 15 min, followed by 40 cycles of 95°C for 20 s and 62°C for 1 min for miR-30d. In addition, expression level of* Beclin-1, bcl2, ULK1* and *LC3* was measured by SYBER qRT-PCR. Each reaction contains 10 μl SYBR master mix, 5 pmol of specific forward and reverse primers and 400 ng of cDNA in total volume of 20μl reaction. The samples were analyzed in duplicates and performed in MicroAmp optical 96-well plate. GAPDH was used as a reference gene using ABI software (StepOne plus, Applied Biosystems). The thermal cycling conditions were as follows: initial denaturation at 95°C for 15 min, followed by 40 cycles of 95°C for 15-30 s and 60°C for 1 min. Moreover, the efficiency of amplification for each miRNA and target gene was assessed by serial dilution of cDNA. 

DNA methylation of Beclin-1, bcl2, LC3 and ULK1

Methylation specific- high resolution melting (MS-HRM) method was exploited for methylation analysis. Gene-specific primers for CpG islands of *Beclin-1, bcl2, LC3* and *ULK1* genes were designed using Generunner and Zymoresearch softwares (Table 4). DNA from NSCLC and paired normal adjacent tissues was extracted using QIAamp DNA extraction kit (Qiagen, United States). Then DNA was quantified and used for bisulfite conversion with the EZ DNA Methylation Gold (Zymo research, USA) in order to perform DNA methylation of *Beclin-1, bcl2, LC3* and *ULK1* genes. The thermal cycling conditions were as follows: preliminary denaturation at 98°C for 10 min followed by 64°C for 2.5 hours and then stored at 4°C up to 20 hours. In addition M.SssI enzyme (Thermo Fisher Scientific, USA) was used to produce fully methylated DNA control. This bisulfite converted DNA was then used for the MS-HRM assay. In brief, 4 μl 5x HOT FIREPol EvaGreen HRM Mix, 6 pmol forward and reverse primers and 1 μl of bisulfite-treated DNA in total volume of 20μl reaction was performed. 

*Data collection and statistical analysis*

The expression level of *miRNA* and target genes were measured by using the comparative 2^−ΔΔCT^ (fold change) method. The statistical significance of relative changes in *miRNA* and *mRNA* expression between different groups of lung tumors were determined by t-test. P-value of < 0.05 was considered as statistically significant. The graphs were created by GraphPad PRISM 5.0 software. According to statistical analysis for DNA methylation, Fisher’s exact test and IBM SPSS Statistics 21 software was determined by t-test.

## Results

*Expression level of miR-20a, miR-30d and miR-204-5p and target genes in tissue samples*

**Figure 1:**
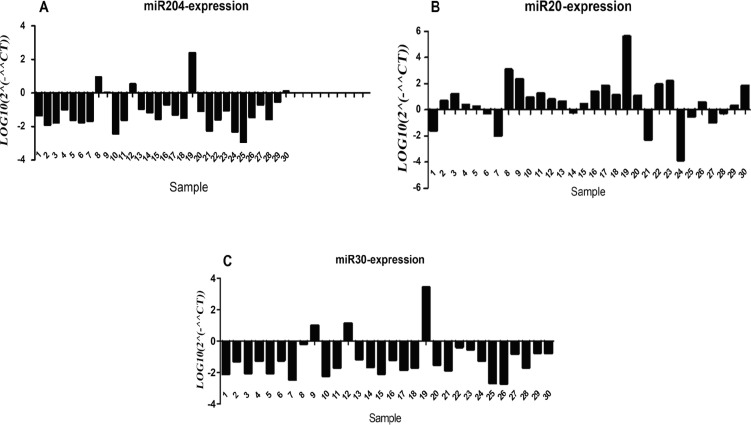
The Expression Level of *miR-204-5p* (A),* miR-20a* (B), and *miR-30d* (C) in NSCLC Patients Compared to Normal Adjacent Tissue. *miR-204-5p* expression was significantly decreased (P=0.048). No significant was found in the expression of *miR-20a* (P= 0.5). *miR-30d *expression level in NSCLC patients compared to normal adjacent tissue was significantly decreased (P= 0.01).

**Figure 2:**
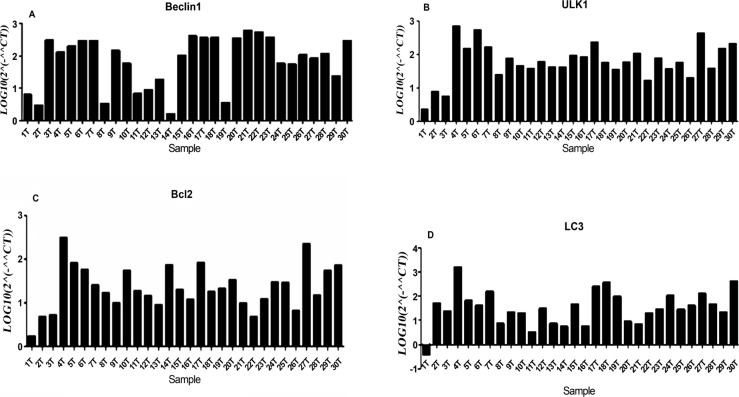
The Expression Level of *Beclin1*, *ULK, bcl2*, and *LC3 *in NSCLC Patients Compared to Normal Adjacent Tissue. (A) Beclin1 expression was significantly increased in NSCLC samples compared to normal adjacent tissues (p-value <0.0001). (B) up-regulation of *ULK1* was found in patients with NSCLC compared to normal adjacent tissue. (p-value <0.0001). (C) *bcl2* expression was significantly increased in NSCLC samples compared to normal adjacent tissues (p-value <0.0001). (D) LC3 expression was significantly increased in NSCLC samples compared to normal adjacent tissue (p-value <0.0001).

In this study, we measured the expression level of *miR-20a, miR-30d *and *miR-204-5p* as well as their target genes *(Beclin1, ULK1, bcl2* and *LC3*) in 30 Iranian patients with NSCLC tumoral samples compared to normal adjacent tissues. The target genes were selected by Targetscan and miRwalk databases according to their high score and more complementary base pairing between miRNAs seed region and 3’UTR sequence of mRNA of target genes. ULK1 was assigned as *miR-20a* target gene and *Beclin1* was chosen as *miR-30d* target gene, while *bcl2* as well as *LC3* were selected as* miR-204-5p *target genes. According to our data obtained in this study, downregulation of miR-30d (P= 0.01, Figure 1C) and *miR-204-5p* (P=0.048, Figure 1A) have been detected in NSCLC patients compared to normal adjacent tissues, while the expression level of* miR-20a* has not been changed significantly (P= 0.5, Figure.1B). Moreover, we found that the expression level of *Beclin1, ULK1, bcl2 *and *LC3* were significantly increased in NSCLC patients (P < 0.0001, Figure 2).

*Correlation of miRNAs expression level with clinical and pathological features in NSCLC patients*

**Figure 3:**
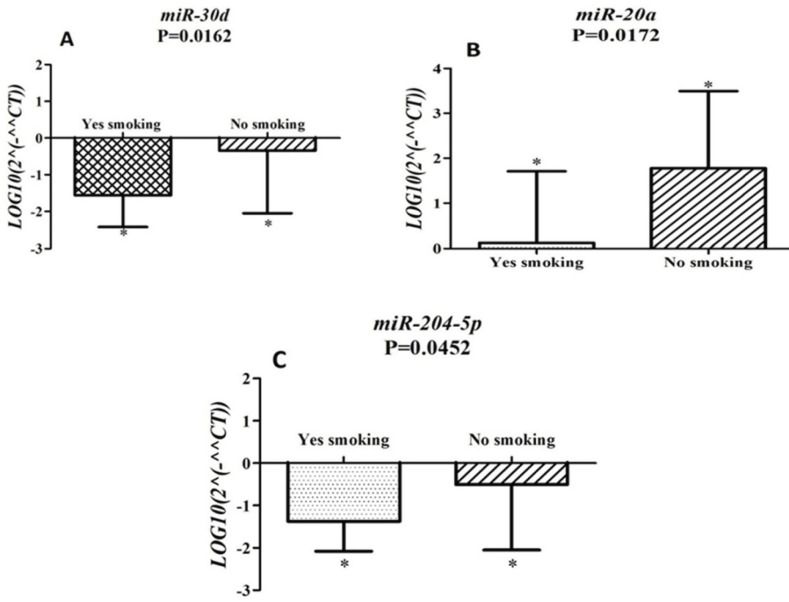
(A) Correlation of miR-30d expression level and smoking. Downregulation of miR-30d expression is correlated with smoking (P= 0.0162). (B) Correlation of miR-20a expression level and smoking. Up-regulation of miR-20a is associated with smoking (P=0.0172). (C) Correlation of miR-204-5p expression pattern and smoking. Downregulation of miR-204-5p expression is correlated with smoking (P=0.0452

In this study, the expression level of *miR-20a, miR-30d* and *miR-204-5p* were compared with subtype (adenocarcinoma and SCC), stage, lymph node involvement and smoking. According to data analyzed, the correlation between overexpression of *miR-20a *(P=0.0172, Figure 3B) with smoking was detected. Moreover, we found the correlation between down-regulation of miR-30d (P= 0.0162, Figure 3A) and miR-204-5p (P=0.0452, Figure.3C) with smoking. According to our data, expression level of these miRNAs with other clinical and pathological features did not show significant correlation. 

*Correlation of target genes expression level with clinical and pathological features in NSCLC patients*

**Figure 4:**
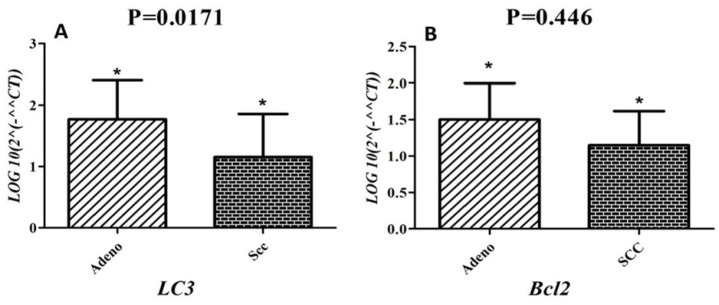
(A) *LC3* expression was increased in adenocarcinoma subtype compared to squamous cell subtype (SCC) (p=0.0171). (B) Up-regulation of *bcl2* expression in adenocarcinoma subtype compared to squamous cell subtype (SCC) patients (p=0.446).

In our experiment, we analyzed correlation between the expression pattern of *Beclin1, ULK1, bcl2 *and *LC3 *with clinical and pathological features (subtype, stage, lymph node metastasis and smoking.). According to data analyzed, overexpression of *LC3* (P=0.0171, Figure.4A) was detected in adenocarcinoma subtype compared to SCC subtype, although the overexpression of *bcl2* in adenocarcinoma compared with SCC subtype was less consistent (P= 0.446, Figure 4B). According to our data, expression level of these genes with other clinical and pathological features did not show any significant correlation. 

*Beclin-1, bcl2, LC3 and ULK1 methylation patterns in NSCLC patients*

**Figure 5:**
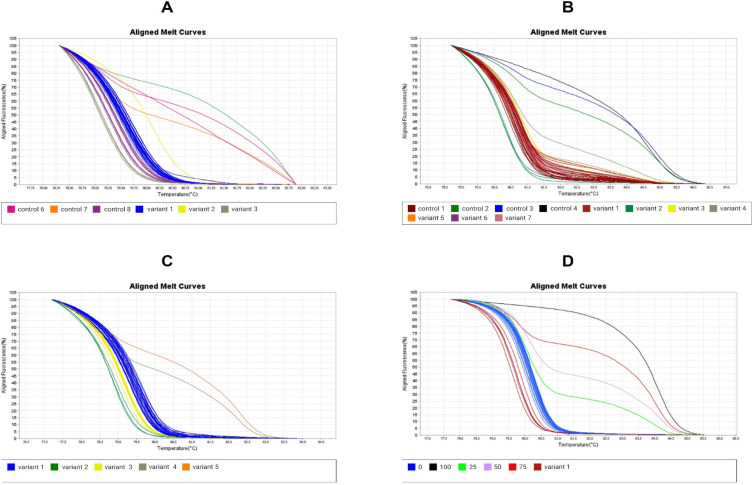
*LC3 *and *ULK1* Promoters were Methylated in 0-12.5% of NSCLC Samples (A & B). 0-25% of methylation was detected in* bcl2 *promoter of NSCLC samples (C). *Beclin_1* promoter was unmethylated in NSCLC samples (D).

Difference in methylation levels of *Beclin-1, bcl2, LC3 *and *ULK1* genes in tumor samples compared to normal tissues were analyzed. According to data obtained, methylation rate of 0-12.5% in LC3 and ULK1 promoters as well as 0-25% in bcl2 promoter was detected (Figure.5), however by IBM SPSS Statistics 21 and Fisher’s exact test, there is no significant correlation in methylation pattern of these genes in NSCLC samples and normal tissues. Regarding to the data analysis, Beclin-1 promoter was unmethylated in NSCLC tissues (Figure.5) and there is no significant correlation in NSCLC samples compared to normal tissues. In addition, a significant correlation was detected in methylation pattern of LC3 and bcl2 promoters by Spearman’s Rho test.

**Table 1:** The Clinico- Pathological Features of NSCLC Patients TNM, Tumor-Node-Metastasis

Tissue	Case
Age	
< 60	14
≥ 60	16
Sex	
Male	23
Female	7
TNM	
I or II	20
III	10
Subtype	
Adenocarcinoma	18
SCC	12
Pack/year	
-	21
4-120	9
Lymph node metastasis	
Yes	13
No	17

**Table 2:** Primer Sequences for Expression Analysis of Target Genes

Ref Seq Accession	Gene Target		Product size (bp)
NM_003766.4	*Beclin-1*	Forward	190
		Reverse	
NM_000633.2	*bcl2*	Forward	136
		Reverse	
NM_003565.2	*ULK1*	Forward	136
		Reverse	
NM_181509.2	*LC3*	Forward	193
		Reverse	
NM_001256799.2	GAPDH	Forward	112
		Reverse	

**Table 3:** Primer Sequences and Stemloops for Quantification of miRNAs

Primer Name	Primer sequence
miR-30d-F	5′ GCGTCCCTGTAAACATC 3′
miR-30d-R	5′ GTATCCAGAGCAGGGTCC 3′
miR-30d Stemloop	5′GGTCGTATGCAGAGCAGGGTCCGAGGTATCCATCGCACGCATCGCACTGCATACGCTTCCA3′
miR-20a-F	5′GCGTCCCTAAAGTGCTTATAG3′
miR-20a-R	5′GTATCCAGAGCAGGGTCC3′
miR-20a Stemloop	5′GGTCGTATGCAGAGCAGGGTCCGAGGTATCCATCGCACGCATCGCACTGCATACGCTACCT3′
miR-204-5p-F	5′ CTTCCCTTTGTCTTCCTATGC 3′
miR-204-5p-R	5′GTATCCAGAGCAGGGTCC 3′
miR-204-5p Stemloop	5′GGTCGTATGCAGAGCAGGGTCCGAGGTATCCATCGCACGCATCGCACTGCATACGAGGCAT3′
RNU44-F	5´ CCT GGA TGA TGA TAG CAA ATG 3´
RNU44-R	5´ TCG TAT CCA GTG CAG GG 3´
RNU44 Stemloop	5´GTCGTATCCAGTGCAGGGTCCGACCGGTATTCGCACTGGATACGACAGTCAG3′

**Table 4:** Primer Sequences for DNA Methylation Assessment of Genes Related to Autophagy

Primer Name	Primer sequence
Beclin-1- F	5′ GTGAGTTTGTGGATTAGGAGTTTTG 3′
Beclin-1-R	5′CGAAACGAAACCTCCAAAACTAC 3′
bcl2-F	5′ GGTCGTGGTAGGTTTGGAAATT 3′
bcl2-R	5′ AACTCCGAACAACGCCAAATA 3′
LC3-F	5′ CACGAACGCCTATCTCTACAA 3′
LC3-R	5′ GATGTCGGGGTAGTAAGTGAT 3′
ULK-1-F	5´ TCGTGTGTTTGTTGATATTGTTTT 3′
ULK-1-R	5´CCTCGACCCTAACTACCAA3′

## Discussion

Autophagy as a conserved pathway contributes in cellular hemostasis and has a fundamental role in physiologic and pathologic mechanism including cancer. In recent years the role miRNA genes in autophagy regulation has been proposed. As around  90% of protein-coding genes are regulated by miRNAs, these elements have an important role in the regulation of various physiologic and pathogenic cellular processes including tumorigenesis and invasion (Hannafon et al., 2019). In this study, the expression pattern of *miR-20a, miR-30d* and *miR-204-5p* as well as their target genes (*Beclin1, ULK1, bcl2* and *LC3*) in NSCLC tumoral cells compared with normal adjacent tissues was investigated. According to our data, *miR-30d *and *miR-204-5p* expression were significantly decreased in NSCLC patients, however changes in the expression pattern of *miR-20a* in NSCLC samples compared to normal tissues has not been significant. In addition, the expression level of *Beclin1, ULK1, bcl2* and *LC3* were significantly increased in NSCLC patients. These results may show a significant role of miR-30d and miR-204-5p in the development of NSCLC. According to some previous studies, miR-30d is found to be capable of suppressing NSCLC cell growth (Tang et al., 2019), indeed this miRNA can act as a tumor suppressor in NSCLC. Moreover, it has been demonstrated that *miR-30d *expression increased in hepatocellular carcinoma (HCC), and its expression is highly associated with the intrahepatic metastasis of HCC (Yao et al., 2010). Therefore, *miR-30d* can have a dual function as a tumor suppressor and oncogene in different kind of cancers. According to some studies, *miR-204* expression was decreased in NSCLC tumoral samples compared with non-cancerous tissue-derived controls. Moreover, *miR-204* silencing in NSCLC cell lines promoted proliferation and cell invasion. Thus, this microRNA may be involved in the NSCLC development (Xia et al., 2014). In addition, it has been indicated that *miR-204-5p* expression was down-regulated in cancerous hepatocellular carcinoma (HCC) tissues compared to adjacent normal tissues and Kaplan–Meier survival curve analysis also demonstrated that down-regulation of *miR-204-5p* predicted worse outcomes of HCC patients. Moreover, it has been showed that ectopic expression of *miR-204-5p* in HCC cell lines inhibited cell proliferation, while the prohibition of miR-204-5p enhanced proliferation (Chu et al., 2018). Recent preliminary studies reported that miR-204-5p was inhibited in colorectal cancer and miR-204-5p can decrease cell proliferation and chemo resistance of colorectal cancer cells (Bian et al., 2016).Thus, miR-204-5p act as a tumor suppressor in different kinds of cancers. 

In this study, the expression level of *Beclin1, ULK1, bcl2* and *LC3* were significantly increased in NSCLC patients compared to normal tissues. Recent studies indicated that the expression of *Beclin1* and* mTOR* were well correlated with survival and clinical stages of human non-small cell lung cancer (NSCLC) patients. Indeed, IL-7 activates PI3 K/Akt/mTOR signaling pathway via Beclin1 to regulate autophagy in lung cancer cells (Jian et al., 2019). What is more, it has been reported that Beclin1 was up-regulated in 67.7% of NSCLC patients, and was suggested that high *Beclin-1* expression predicts longer survival in locally advanced NSCLC (Lee et al., 2019). Thus, this gene may act as an oncogene which is related to longer survival in patients with NSCLC. In our study, ULK1 was significantly over expressed in NSCLC samples. In a recent study was demonstrated that down-regulation of Ulk1 suppresses NSCLC cell growth and sensitizes NSCLC cells to cisplatin by modulating both apoptosis and autophagy pathways (Tang et al., 2017). In addition, it has been indicated miR 21 leads to autophagy related AMPK/ULK1 signaling pathway activation in NSCLC cell lines, while over expression of *ULK1* reversed the biological functions of miR 21. Indeed, miR 21 regulates autophagy activity by AMPK/ULK1 signaling pathway, and increases the proliferation, migration and invasion of NSCLC cells (Li et al., 2018). According to these data, ULK1 may act as an oncogene and tumor suppressor gene and this factor might be a promising target for NSCLC treatment.* Bcl2* and* LC3* are two more factors which were analyzed in this study. According to the data obtained, the overexpression of these markers was found in NSCLC patients compared to normal samples. In recent studies, overexpression of *bcl2* was observed in lung cancer cells (Ma et al., 2019). In other study showed that *bcl2* expression and *bcl2/Bax* expression level might be beneficial as independent diagnostic factors in lung carcinoids. Indeed, therapeutic approaches using down-regulation of *bcl2* might be useful in lung cancer (Velinovic et al., 2019). In a recent study, investigated sh-UCA1 significantly inhibited the protein levels of *Beclin1* and *LC3-* and the cell growth and autophagy was decreased by down-regulated *LC3 *expression (Yang et al., 2019). Although some studies show higher expression of *LC3* is correlated with higher growth for tumor cells, this indicated the autophagy markers* LC3* and *p62* are differentially expressed in NSCLC samples and high *LC3* expression seem to be linked to lower tumor aggressiveness, while high *p62* expression was significantly associated with aggressive tumor behavior (Schläfli et al., 2016). According to mentioned studies, LC3 may act as both oncogene and tumor suppressor gene in NSCLC. In this study, methylation of *bcl2, LC3, ULK1 *and *Beclin1 *were also analyzed and according to our data, *bcl2* gene was methylated in NSCLC samples compared to adjacent normal tissues, however, our data did not show a significant change about methylation of this gene. In recent studies, it has been indicated high expression levels of *p53* and *CHGA* were correlated with the methylation of *bcl2 *in NSCLC samples as well as methylation of *bcl2, RARB* and *SIX6* was associated with smoking. Moreover, they found methylation frequencies of 7 genes including bcl2 were significantly higher in stage I NSCLC than in non-cancerous lung diseases (Zhao et al., 2013). In another study demonstrated that bcl2 was hypo-methylated in NSCLC samples (Lokk et al., 2012). Indeed, methylation of this gene may have an essential impact on lung cancer progression. In this study, there was not any significant difference in the methylation pattern of LC3 in NSCLC patients compared to normal samples. However, some others indicated LC3Av1 was inactivated at the transcriptional level due to aberrant DNA methylation in esophageal squamous cell carcinoma (ESCC) cell lines and primary tumors. This result suggests that LC3Av1 functions may be pivotal in carcinogenesis (Bai et al., 2012). In our study, methylation of ULK1 was analyzed in tumoral samples compared with normal tissues and no significant difference was detected in NSCLC samples in comparison to normal tissues. In a study demonstrated that hyper-methylation of ULK2 promoter was detected by bisulfite sequencing in glioma cell lines. Indeed, ULK2 promoter methylation and transcript levels showed significant negative correlation. These data suggested that inhibition of autophagy via ULK1 down-regulation has a pivotal role for glioma development. *Beclin1* was another factor which its methylation was analyzed in NSCLC compared to normal tissues. In our study, no methylation was occurred and expression pattern of this gene was increased significantly. Another study indicated TCF21 knockdown cells showed significantly upregulated *ATG-9, Beclin1 *and *LC3-I/II* expressions. Inhibition of autophagy by 3-methyladenine (3-MA) elevated* TCF21* expression and increased cell apoptosis (Chen et al., 2018). Moreover, it has been analyzed miR-129-5p methylation and they found out hyper-methylation of this microRNA was associated with overexpression of *Beclin1* in intervertebral disc degeneration (Zhao et al., 2017b). 

In conclusion, *miR-30d *and *miR-204-5*p expression levels were decreased significantly and *Beclin1, ULK1, bcl2* and *LC3* expression were significantly increased in NSCLC patients compared to normal adjacent tissues. These results suggesting that mentioned microRNAs might act as tumor suppressor genes, however, target genes might act as oncogenes in NSCLC samples. In addition, our data about methylation pattern of target genes did not show a significant change in tumoral samples comparing to adjacent normal tissues.

## Author Contribution Statement

Study conception and design: Morteza Karimipoor, Shohreh Zare Karizi. Acquisition of data: Minoo Pargol, Shima Zare Karizi, Masoumeh Akbari, Mohammad Behgam Shadmehr. Analysis and interpretation of data:Minoo Pargol, Shima Zare Karizi, Masoumeh Akbar. Drafting of manuscript:Bahareh Nourmohammadi. Critical revision: Morteza Karimipoor, Shohreh Zare Karizi. The first three authors equally contributed to this work.
